# The influence of beam optics asymmetric distribution on dose in scanning carbon‐ion radiotherapy

**DOI:** 10.1002/acm2.13656

**Published:** 2022-05-30

**Authors:** Sixue Dong, Fuquan Zhang, Nicki Schlegel, Weiwei Wang, Jiayao Sun, Yinxiangzi Sheng, Xiaobin Xia

**Affiliations:** ^1^ Shanghai Institute of Applied Physics Chinese Academy of Sciences Shanghai 201800 China; ^2^ University of Chinese Academy of Sciences Beijing 100049 China; ^3^ College of Physical Science and Technology (College of Nuclear Science and Engineering) Sichuan University Chengdu China; ^4^ Department of Nuclear Medicine, Shanghai Proton and Heavy Ion Center Fudan University Cancer Hospital Shanghai China; ^5^ Department of Medical Physics Shanghai Proton and Heavy Ion Center Shanghai China; ^6^ Shanghai Key Laboratory of Radiation Oncology (20dz2261000) Shanghai China; ^7^ Shanghai Engineering Research Center of Proton and Heavy Ion Radiation Therapy Shanghai China

**Keywords:** beam optics asymmetry, Monte Carlo simulation, relative dose comparison, scanning carbon‐ion radiotherapy

## Abstract

**Purpose:**

To quantify the influence of beam optics asymmetric distribution on dose.

**Methods:**

Nine reference cubic targets and corresponding plans with modulation widths (M) of 3, 6, and 9 cm and with center depths (CDs) of 6, 12, and 24 cm were generated by the treatment planning system (TPS). The Monte Carlo code FLUKA was used for simulating the dose distribution from the aforementioned original plans and the dose perturbation by varying ±5%, ±15%, ±20%, ±25%, and ±40% in spot full width half maximum to the *X*‐direction while keeping consistent in the *Y*‐direction. The dosimetric comparisons in dose deviation, *γ‐*index analysis, lateral penumbra, and flatness were evaluated.

**Results:**

The largest 3D absolute mean deviation was 15.0% ± 20.9% (mean ± standard deviation) in M3CD6, whereas with the variation from −15% to +20%, the values were below 5% for all cube plans. The lowest 2D *γ*‐index passing rate was 80.6% with criteria of 2%–2 mm by a +40% variation in M3CD6. For the M9CD24 with a −40% variation, the maximum 1D dose deviations were 5.6% and 15.7% in the high‐dose region and the edge of the radiation field, respectively. The maximum deviations of penumbra and flatness were 3.4 mm and 11.4%, respectively.

**Conclusions:**

The scenario of beam optics asymmetric showed relatively slight influence on the global dose distribution but severely affected dose on the edge of the radiation field. For scanning carbon‐ion therapy facilities, beam spot lateral profile settings in TPS base data should be properly handled when beam optics asymmetry variation is over 15%.

## 1 INTRODUCTION

Carbon‐ion radiation therapy (CIRT) has physical and biological advantages compared to proton radiotherapy. Carbon‐ion beams possess smaller spot sizes and steeper dose gradients, reducing the absorbed dose to organs‐at‐risk consequently.^1^ Beyond dosimetric advantages, CIRT has demonstrated superiority in treating low *α*/*β* ratio tumors, hypoxic tumors, and tumors with a high risk of metastasis.^2^ With a spot scanning technique, the carbon‐ion beam requires periodical quality assurance (QA) of spot characteristics to ensure the precision and stability of CIRT.

The spot‐related commissioning and QA parameters for proton beams were recommended in AAPM TG224.^3^ It mainly includes spot size, spot position, and spot shape. Kraan et al.^1^, ^4^, ^5^ proposed that dose distributions proved robust against stochastic spot charge perturbation and then summarized the effects of changes in different spot parameters such as spot size and spot spacing on planning and delivering systematically. Parodi et al.^6^ investigated the beam asymmetric distribution by using truncated Gaussian beam profiles and reported that an obviously large focus size (above 150%–200% of the nominal value) must be prevented. For spot size variation, AAPM TG224^3^ reported ±10% as the spot size QA tolerance of proton beams. Both Widesott et al.^7^ and Xing et al.^8^ performed related research on spot spacing and reported that 2.5 is the appropriate ratio of spot size (full width half maximum, FWHM) to spot spacing for static tumors. Yu et al.^9^ built an analytic tool to simulate the impact of spot position error in intensity‐modulated proton therapy.

Compared to proton beams, the more substantial magnetic rigidity of carbon‐ion beams leads to more difficulties in the construction of the gantry and the commission of the magnets.^10^ Such difficulties might lead to beam optics asymmetric distribution. Kleffner et al.^11^ reported a trapezoid‐like spot shape in the *X*‐direction due to the horizontal extraction process in Heidelberg Ion Therapy Facility (HIT, Germany). In Heavy Ion Medical Accelerator in Chiba (HIMAC, Japan), Furukawa et al.^12^ have proposed a method to compensate for the asymmetric distribution by using a thin scatter. However, the spot size will be enlarged by scattering. Moreover, it is unlikely to overcome beam‐emittance asymmetry issues fully.^13^ In the clinically implemented carbon‐ion treatment planning system (TPS) Syngo (SIEMENS, Germany), spot sizes in *X*‐ and *Y*‐direction were assumed to be completely concordant. A similar beam spot setting was applied in RayStation (RaySearch Laboratories, Stockholm/Sweden) during the commissioning of a proton facility.^14^ Nevertheless, the spot size ratio in the horizontal versus vertical directions at the isocenter may be different and change over time. Therefore, it is indispensable to evaluate the dosimetric impact induced by beam spot asymmetry.

Klodowska et al.^15^ investigated the impact of proton beam optics asymmetry at a spot rotation of 45° and reported that the flatness would be lower than 5% with a +50% asymmetry. However, their study only focused on one cube target of a single size (3 × 3 × 3 cm^3^) and ignored the impact of the target depth. Moreover, taking flatness as the only parameter for analysis might be insufficient. To the best of our knowledge, most literature reported research on spot size variation, but few for the beam optics asymmetry and its characteristics in both spot scanning proton and carbon‐ion beams. Meanwhile, as far as our clinical experience and the extensive literature, check of beam optics asymmetry was not included in the acceptance test procedures of carbon beam facilities.^16^, ^17^ In this study, cube targets with different volumes and/or depths in water and corresponding plans were generated by using TPS. Such plans were implemented in Monte Carlo code FLUKA.^18^ We kept the spot size consistent in the *Y*‐direction while changing the spot size in the *X*‐direction. The dose distribution of original plans, as well as the plans with various spot sizes in the *X*‐direction, were simulated by using FLUKA. 3D absolute mean point‐to‐point dose deviation, *γ‐*index analysis for 2D dose distribution, 1D flatness, and penumbra were analyzed. Consequently, the asymmetric relevance of beam optics impact on the quality of scanning carbon‐ion beam dose distribution was discussed.

## 2 METHODS

### 2.1 Targets and plans generated by TPS

Cubic targets with different range modulation were modeled in Syngo TPS (VC13C, SIEMENS, Germany). Three cubes with different dimensions (3, 6, and 9 cm) were generated to make a thorough inquiry into the effect of beam optics asymmetry on dose distributions under the various sizes of tumors. The cube centers were placed in a water phantom at different locations (6, 12, and 24 cm). The character M represents the modulation width of the cube, whereas the character “CD” represents the center depth. For example, M3CD6 represents a 3 × 3 × 3‐cm^3^ cube located at 6‐cm depth in a water phantom.

The plans were generated by Syngo TPS. The absorbed dose calculation algorithm of Syngo TPS is based on the pencil beam algorithm. The lateral profile distribution is modeled as a two‐dimensional symmetric Gaussian, and the double‐Gaussian scattering model describes lateral fluence distribution.^19^ There are five levels of spot sizes in each energy of carbon‐ion beam commissioning in the TPS. The FWHM values closest to and greater than 6 mm were chosen for optimization in each plan (FWHM varying from 6.1 to 8.1 mm for all plans). The spot spacing was set to 2 mm and held constant in all the plans. The ratio of spot FWHM to grid size was greater than 3.^8^ A 3‐mm range step was chosen. The prescribed absorbed dose was set to 1 Gy. Plans were optimized to achieve a target homogeneity of less than 3%.^8^ Subsequently, nine cube plans were generated, and the DICOM RTplan was obtained for further use.

### 2.2 Beam optics modeling in Monte Carlo

In this study, the Monte Carlo code FLUKA (4‐1.1, CERN) was chosen as a simulation software because of its widespread usage and its accuracy in the simulation of particle beam radiotherapy.^18^, ^20^ The default setting of “HADROTHErapy” was used in the FLUKA to simulate carbon‐ion beams transport.^21^, ^22^ The thermal neutron transport threshold was set at 10^−5^ eV, and the transport threshold of other particles was set at 100 keV.^18^

In‐line with the previous work of Sheng et al.,^22^ the carbon‐ion beam model was established by using FLUKA before embarking on this study. The geometric structure of the 1D‐ripple filter and range shifter was built, and we assumed a parallel beam without considering the beam angular distribution in the simulation, according to Parodi et al.^21^ The spot size was implemented in the FLUKA source code as a Gaussian shape. The dose distributions of three target cubes (M3CD6, M6CD12, and M9CD24) calculated by TPS were used to compare with the simulated results in FLUKA. The relevant results of comparison and verification were shown in the Supplementary Materials. Moreover, 3D absorbed doses were scored with a 2‐mm grid in all directions in this work.

### 2.3 Variations of the beam optics symmetry

The DICOM RTplans of nine cubes derived from TPS were converted to FLUKA source files. The original beam optics parameters from the RTplans were imported to FLUKA, and the dose distributions of the original plan simulated from FLUKA were utilized as references. FWHM variations (±5%, ±15%, ±20%, ±25%, and ±40%) were applied to the *X*‐direction, whereas it was consistent in the *Y*‐direction.

### 2.4 Data analysis

Absolute mean point‐to‐point dose deviation was chosen to analyze the 3D dose distributions. The dose region higher than 10% of the maximum dose was selected to calculate the 3D dose deviations by using the following formula^23^:

(1) $$\begin{array}{c} D_{Dev}\;\left(\%\right)=\frac{\;\sum_{i=1}^{N}\left(\frac{\left|d_{i}-dref_{i}\right|}{dref_{i}}\right)}{N}\; \end{array}$$

where *D_Dev_* (%) was the absolute mean point‐to‐point dose deviation, *d* and *dref* were the perturbed and reference doses, respectively, *i* was the corresponding scoring point, and *N* was the total points of the scoring region.

The *γ‐*index analysis tool (VeriSoft 7.1, PTW—Freiburg, Germany) was chosen to analyze the 2D dose distribution in the *X*–*Y* plane of the target center. The parameters of the *γ* analysis were as follows: 2%–2 mm, the negligible threshold dose was 10% and the normalization of *γ* analysis was performed on the global dose maximum.^24^, ^25^, ^26^, ^27^ 1D dose distributions in the central *X*‐direction of the target center were also compared.

In addition, lateral penumbra and 1D flatness along the *X*‐axis of the *X*–*Y* plane at the target center were also analyzed.^3^ The lateral penumbra was defined by the distance between 80% and 20% of the prescribed dose. The flatness was calculated by using the following formula.^17^ The calculation area (the so‐called target width in ICRU report 78^17^) was defined as the distance between two lateral penumbra widths from the 50% isodose levels of the lateral‐beam profile.

(2) $$\begin{array}{c} Flatness\;:F_{LP}\;\left[\%\right]=\;\frac{D_{LP\;max\;}-D_{LP\;min}}{D_{LP\;max}+D_{LP\;min}}\;\times 100 \end{array}$$

where *D*_LPmax_ and *D*_LPmin_ are the maximum and minimum absorbed dose, respectively.

In the following sections, the high‐dose region was defined as the area where the absorbed dose exceeded 90% of the prescribed dose, whereas the area between the high‐dose region and the 80% of the prescribed dose was defined as the edge of the radiation field.

## 3 RESULTS

### 3.1 Dose deviation and 2D gamma passing rate (*γ*‐PR)

The 3D absolute mean point‐to‐point dose deviations are shown in Table 1. From the overall 3D dose deviations, the best and worst cube series results were M9 and M3. By a decrease of 40% on the spot size in the *X*‐direction, the 3D dose deviation was 15.0% ± 20.9% (mean ± standard deviation, SD) in M3CD6, and 3.4% ± 9.0% in M9CD24. With the variation from −15% to +20%, 3D dose deviations were below 5% for all cube plans.

**TABLE 1:** 3D absolute mean point‐to‐point dose deviation for all the cube groups (mean ± standard deviation)

**Variations (%)**	**M3CD6 (%)**	**M3CD12 (%)**	**M3CD24 (%)**
‐40	15.0 ± 20.9	13.3 ± 18.1	12.5 ± 16.1
‐25	8.0 ± 12.9	7.4 ± 10.7	6.9 ± 9.8
‐20	6.2 ± 10.2	5.6 ± 8.4	5.4 ± 7.7
‐15	4.5 ± 7.5	4.0 ± 6.1	3.7 ± 5.8
‐5	1.5 ± 2.4	1.4 ± 1.8	1.2 ± 1.8
5	1.4 ± 2.2	1.2 ± 1.8	1.1 ± 1.7
15	3.6 ± 6.2	3.1 ± 5.0	2.8 ± 4.8
20	4.7 ± 8.0	3.9 ± 6.3	3.6 ± 6.2
25	5.7 ± 9.7	4.7 ± 7.7	4.3 ± 7.6
40	8.3 ± 14.0	6.7 ± 10.9	6.1 ± 11.0

**Variations (%)**	**M6CD6 (%)**	**M6CD12 (%)**	**M6CD24 (%)**
−40	8.9 ± 17.3	9.0 ± 15.8	7.4 ± 13.5
−25	5.3 ± 10.7	5.2 ± 9.9	4.6 ± 8.2
−20	4.2 ± 8.4	4.1 ± 7.8	3.7 ± 6.5
−15	3.1 ± 6.2	2.9 ± 5.7	2.4 ± 4.7
−5	1.3 ± 2.0	1.2 ± 1.8	1.1 ± 1.5
5	1.2 ± 2.0	1.3 ± 1.8	1.5 ± 1.7
15	2.7 ± 5.5	2.4 ± 5.1	2.5 ± 3.9
20	3.4 ± 7.2	2.9 ± 6.6	3.0 ± 5.1
25	4.0 ± 8.7	3.5 ± 7.9	3.4 ± 6.2
40	5.8 ± 12.9	5.1 ± 11.7	4.0 ± 9.4

**Variations (%)**	**M9CD6 (%)**	**M9CD12 (%)**	**M9CD24 (%)**
−40	6.3 ± 13.2	5.6 ± 12.7	5.8 ± 11.4
−25	3.8 ± 7.9	3.6 ± 7.9	3.7 ± 7.0
−20	3.1 ± 6.2	3.0 ± 6.2	2.7 ± 5.7
−15	2.5 ± 4.5	2.2 ± 4.5	2.1 ± 4.2
−5	1.3 ± 1.6	1.2 ± 1.5	1.6 ± 1.7
5	1.2 ± 1.5	1.4 ± 1.6	1.1 ± 1.4
15	2.1 ± 3.9	2.2 ± 4.0	1.9 ± 3.8
20	2.4 ± 4.9	2.6 ± 5.1	2.2 ± 4.9
25	2.8 ± 6.0	3.0 ± 6.2	2.5 ± 6.0
40	3.8 ± 8.8	3.9 ± 9.2	3.4 ± 9.0

The *γ*‐index passing rates (*γ*‐PRs) of the nine different cubes with criteria of 2%–2 mm were shown in Table 2. From the overall 2D *γ*‐PRs, similar to the results of 3D dose deviations, the best and worst cube groups were M9CD24 and M3CD6, respectively. With an increase of 40% on the spot size in the *X*‐direction in M3CD6, the *γ*‐PR was 80.6%, whereas all *γ*‐PRs could achieve 90.0% with the criteria of 2%–2 mm when the spot size in the *X*‐direction varies within ±30%.

**TABLE 2:** 2D *γ*‐PRs with the criteria of 2%–2 mm for all the cube groups

**Variations (%)**	**M3CD6 (%)**	**M3CD12 (%)**	**M3CD24 (%)**	**M6CD6 (%)**	**M6CD12 (%)**	**M6CD24 (%)**	**M9CD6 (%)**	**M9CD12 (%)**	**M9CD24 (%)**
−40	86.9	96.2	100.0	93.4	98.0	97.4	94.7	97.2	99.8
−25	98.2	100.0	100.0	98.0	100.0	98.4	98.6	99.9	100.0
−20	98.2	100.0	100.0	99.3	100.0	98.7	99.9	99.9	100.0
−15	100.0	100.0	100.0	100.0	100.0	98.4	100.0	99.9	100.0
−5	100.0	100.0	100.0	100.0	100.0	98.5	100.0	99.9	100.0
5	100.0	100.0	100.0	100.0	99.9	98.3	99.9	99.7	100.0
15	99.5	97.2	99.8	98.9	98.5	98.2	98.3	98.4	99.9
20	96.5	94.9	99.1	96.6	96.6	98.1	97.0	97.1	99.9
25	93.5	92.7	97.7	94.2	94.4	97.0	95.5	95.9	99.7
40	80.6	86.6	89.6	85.2	89.9	94.2	88.9	92.1	98.9

**FIGURE 1:**
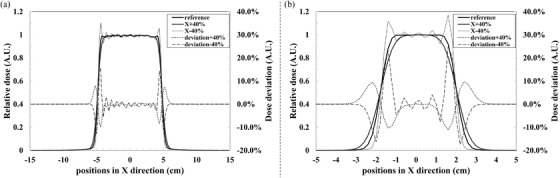
Relative dose (left axis) and dose deviation (right axis) on different positions in the cube M9CD24 (a) and the cube M3CD6 (b). Fine gray line represents 40% increase of spot size in the *X*‐direction, and the bold gray line represents 40% decrease, the black line is the reference. Dotted and dash lines are represented to the deviations of +40% and −40%, respectively

1D relative dose profile and dose deviation in the *X*‐direction at the target center slice of the best (cube M9CD24) and worst (cube M3CD6) match situations are shown in Figure 1a,b, respectively. For the M9CD24 (a), dose ripples can be seen in the high‐dose region and the edge of the radiation field. In the high‐dose region, the maximum deviation was 5.6%, and the mean deviation was −0.4% (1.6%) with −40% variation, whereas the maximum and mean values were 2.8% and −0.3% (0.6%) with +40% variation, respectively. For the edge of the radiation field, the maximum and mean values were −9.8% and −0.8% (6.5%) for a +40% variation versus 15.7% and 0.4% (10.2%) with a −40% variation. In M3CD6 (b), a general worse result (larger dose ripples and a more than 20% maximum deviation) than M9CD24 could be observed clearly.

### 3.2 Flatness and lateral penumbra

**FIGURE 2:**
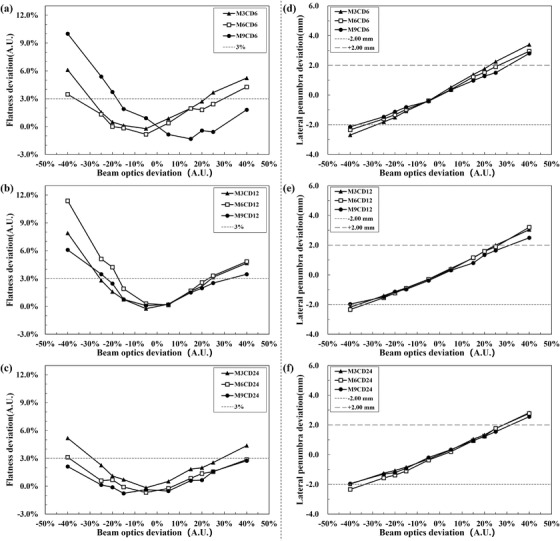
1D flatness (a–c) and lateral penumbra (d–f) deviation relative to the reference cube group in the *X*‐direction of the target center slice. A deviation of 3% for 1D flatness^31^ and that of ±2 mm for lateral penumbra^3^ were displayed as dotted lines

The flatness deviations relative to the reference were shown in graphs a–c of Figure 2. With negative beam optics variation, the worst flatness deviation was found in M6CD12 (11.4%). For positive beam optics variation, the worst case was found in M3CD6 (5.2%). Deviation of lateral penumbra was shown in graphs d–f of Figure 2. The minimum (−2.7 mm) and maximum (3.4 mm) lateral penumbra deviation values were in M3CD6 with the variations of −40% and +40%, respectively.

## 4 DISCUSSION

This study performed Monte Carlo simulations to simulate the beam optics variation in a single direction (*X*‐direction). The impact of beam optics asymmetric distribution in scanning CIRT was evaluated. Apart from 3D dose comparison, we conducted a further analysis of the 1D flatness and penumbra and aimed to obtain the influence precisely for the beam optics asymmetry in CIRT.

Our results demonstrated that the optics asymmetry caused a noticeable influence on the dose distribution. When the variation of beam optics asymmetry was beyond 15%, there would be a risk of 3D mean dose deviation exceeding 5%, and the maximum dose deviation would be more than 20%. As is shown in Table 1, the 3D absolute mean point‐to‐point dose deviations decreased as the target depth in water increased. Moreover, the 2D *γ*‐PRs (Table 2) showed a similar trend, higher *γ*‐PRs were observed for deep targets than for shallow ones. The main reason was the multiple Coulomb scattering of carbon‐ion beams in water, and it could compensate for the effect caused by the change of spot size to some extent.^28^ In addition, another conclusion could be drawn from the result of 3D dose deviations and 2D *γ*‐PRs, and for clinical treatment, better robustness could be observed in large tumor targets than in smaller ones when against the perturbation of beam optics asymmetry.

In terms of the dose distribution in the entire radiation field, Figure 1 shows that the dose distribution in the edge of the radiation field was more affected than the high‐dose region when the optics was asymmetrical. The main reason is more particles were arranged at the edge of the target than in the central region during the process of optimization in TPS to obtain a rapid lateral dose falloff. A positive variation of beam optics asymmetry would cause a less steep dose gradient. It would result in an insufficient dose at the edge of the radiation field, and the maximum dose insufficiency would exceed 10%. Chanrion et al.^29^, ^30^ indicated that a positive variation of spot size would decrease the planning target volume (PTV) coverage, and the underdose would involve almost the entire PTV when the spot size variation is greater or equal to +25%. From our results, the mean dose insufficiency would exceed 5% when the asymmetric variation is larger than +20%, and it further demonstrated that the beam optics asymmetry could also cause the underdose in PTV, similar to what Chanrion reported. It should be mentioned that the ratio of the edge of the radiation field was increasing along with the target size decreasing, and it might cause a worse result of flatness deviation in smaller targets than in larger ones. Moreover, the impact of beam optics asymmetry distribution on lateral penumbra deviation will diminish with increasing target depth in water. In summary, we conclude that tumors of small volume and shallow depth are generally more sensitive to beam optics variation.

1D flatness deviation over 3%^31^ was observed by variating the beam spot in the *X*‐direction beyond −15% for target M6CD12 and M9CD6. Our results show that carbon‐ion beam optics asymmetry variation could affect flatness as well as the absolute dose. The dosimetric impact of beam optics asymmetry needs to be evaluated and properly handled. As recommended in TG224,^3^ spot size, spot position, and spot shape at different energy levels could be detected using an EDR2 film (Carestream Health, Inc., Canada), an ion chamber array, strip chambers, and 2D high‐resolution dosimetry system consisting of a scintillating screen coupled with a charged coupled device (CCD) camera (e.g., Lynx).

During the commissioning of a carbon‐ion facility, when the beam optics asymmetry is beyond 15%, one recommendation is to set different beam spot lateral profiles in TPS base data if it is applicable in the TPS settings. If a circular beam spot in the TPS base data is mandatory, then the mean value of the spot size in *X*‐ and *Y*‐directions could be applied. Spot size should fulfill the criteria recommended in the literature. Take a spot size tolerance of ±10% from TG‐224 for example, if the mean values of spot size in the *X*‐ and *Y*‐directions were applied, beam optics asymmetry beyond 20% would fall the tolerance of spot size, which needs to be fixed. In other words, the possible worst scenario of beam optics asymmetry would be 20% (spot sizes variation of +10% in the *X*‐direction and −10% in the *Y*‐direction). We have simulated and compared the corresponding beam quality of the worst scenario. The 3D absolute mean dose deviation never exceeded 5% and the 2D *γ*‐PRs were never below 99% with criteria of 2%–2 mm. The deviation of flatness and lateral penumbra were within tolerance for all the cubic targets (Table S1 and Figure S4). Once a proper spot size value in the *X*‐ and *Y*‐direction was applied in the base data of TPS, as for the routine QA, the dosimetric impacts on the spot lateral profiles could be estimated by the QA tolerance of spot size recommended in the literature.

One limitation of this study is that the covariance between the orthogonal (*X* and *Y*) directions of the pencil beam propagation was not evaluated. The worst case scenario of spot shape variation could happen when the spots are rotated by 45° with the carbon gantry. This requires further investigation.

The plans generated by TPS in this study were all optimized with a uniform physical dose. The relative biological effectiveness was not considered. Therefore, such a study might have reference significance for proton beams. Because of the larger spot size, a worse dose distribution could be generally expected in proton beams than in carbon‐ion beams with the same perturbation of beam optics asymmetry.^32^

## 5 CONCLUSION

Beam optics asymmetric distribution in scanning carbon‐ion beam was evaluated by using Monte Carlo simulation. Based on analyses of dose deviations, a target with a larger volume and further depth location showed better robustness against perturbation when facing a beam optics asymmetric distribution. The beam optics asymmetry had little effect on the global dose distribution in the high‐dose region but significantly impacted the edge of the radiation field. To obtain a precise analysis of the difference in beam characteristics, conducting 1D lateral penumbra and flatness analyses would be more sensitive to illustrating the influence of dose distribution than analyses of 3D dose deviations and 2D *γ‐*index analysis. We strongly recommended that the impact of beam optics asymmetry on dose distribution should be carefully evaluated during the commissioning of individual carbon‐ion therapy facilities. Beam spot lateral profile settings in TPS base data should be properly handled when beam optics asymmetry variation is over 15%.

## CONFLICT OF INTEREST

The authors have no relevant conflicts of interest to disclose.

## AUTHOR CONTRIBUTION

Sixue Dong: data gathering and analyzing, as well as writing this manuscript. Fuquan Zhang: developing the analytical script program based on Python and MATLAB. Nicki Schlegel, Weiwei Wang, and Jiayao Sun: providing language guidance and making target plans in TPS Syngo. Yinxiangzi Sheng and Xiaobin Xia: devising the main conceptual ideas and project outline, as well as providing writing guidance.

## Supporting information


**Figure S1** FLUKA fit (lines) and TPS (dots) IDDs for carbon‐ion beam energies of 88.65, 171.59, 234.05, 285.35, 351.41, and 430.12 MeV/u
**Figure S2** Sample of the TPS and FLUKA spot sizes in the *X*‐direction for six energies and three positions around the isocenter
**Figure S3** 1D dose profile comparison between the MC simulation (line) and TPS (dot) for M6CD12
**Figure S4** Relative dose (left axis) and dose deviation (right axis) on different positions in the cube M9CD24 (a) and the cube M3CD6 (b)
**Table S1** The results of 3D absolute mean dose deviation, 2D *γ*‐PRs (2%–2 mm), the deviation of 1D flatness and lateral penumbra with spot sizes variation of +10% in the *X*‐direction and −10% in the *Y*‐direction

Supplementary Materials

## Data Availability

The data that support the findings of this study are available from the corresponding author upon reasonable request.
