# Supplementary material for: The influence of beam optics asymmetric distribution on dose in scanning carbon‐ion radiotherapy
Source: J Appl Clin Med Phys. 2022 May 30;23(9):e13656. doi: 10.1002/acm2.13656 (PMC9512340; doi:10.1002/acm2.13656)
Supplement: Supplementary file 1 — Figure S1 FLUKA fit (lines) and TPS (dots) IDDs for carbon‐ion beam energies of 88.65, 171.59, 234.05, 285.35, 351.41, and 430.12 MeV/u Figure S2 Sample of the TPS and FLUKA spot sizes in the X‐direction for six energies and three positions around the isocenter Figure S3 1D dose profile comparison between the MC simulation (line) and TPS (dot) for M6CD12 Figure S4 Relative dose (left axis) and dose deviation (right axis) on different positions in the cube M9CD24 (a) and the cube M3CD6 (b) Table S1 The results of 3D absolute mean dose deviation, 2D γ‐PRs (2%–2 mm), the deviation of 1D flatness and lateral penumbra with spot sizes variation of +10% in the X‐direction and −10% in the Y‐direction [file ACM2-23-e13656-s001.docx]

# Supplementary Material

At the geometry modeling stage in FLUKA, the geometric structure of the 1D-ripple filter (RiFi) and range shifter were built. In line with the clinical devices, the 1D-RiFi was modeled using a periodic groove structure of 1 mm laterally and a thickness of 3 mm, and a 2 cm thickness plate was used to model the structure of the range shifter in FLUKA. Plexiglass also known as polymethyl methacrylate (PMMA, the density is 1.17 g/cm3) was selected as the material for both of them.

TPS Syngo (SIEMENS, Germany) has been used in our center, and there were five degrees of spot size for each energy level. Such values closest to and greater than 6 mm was chosen for optimization in Syngo (FWHM varying from 6.1 mm to 8.1 mm for all plans). During the matching process in FLUKA, we also chose the spot size that closest to and greater than 6 mm for each energy level in the base data of Syngo as the reference spot size.

The matching of monoenergetic carbon ion beam characterization was shown in supplemental figure 1 (Integrate depth dose, IDD) and supplemental figure 2 (Spot FWHM). The differences of range, Bragg peak width and distal falloff width were never exceeded 0.1 mm and the difference of spot FWHM were never exceeded 0.15 mm.

The carbon beam model in FLUKA has been performed by using gamma index analysis, dose distributions of three target cubes (M3CD6, M6CD12, and M9CD24) from FLUKA were compared with the result of TPS and it showed a high γ-PRs (94.7%, 96.3% and 97.1% for M3CD6, M6CD12, and M9CD24 with the criteria of 2%- 2 mm, respectively). The 1D depth and lateral dose profile comparisons of M6CD12 were shown in supplemental figure 3 as examples. The mean point-to-point dose deviation (>10% maximum dose) of 1D depth and lateral dose profiles were 2.89%±10.47% (mean ± SD) and 1.30%±1.79% (mean ± SD).

**Supplemental Fig 1** FLUKA fit (line) and TPS (dot) IDDs for carbon ion beam energies of 88.65 MeV/u, 171.59 MeV/u, 234.05 MeV/u, 285.35 MeV/u, 351.41 MeV/u and, 430.12 MeV/u.

**Supplemental Fig 2** Sample of the TPS and FLUKA spot sizes in X direction for six energies and three positions around the isocenter.


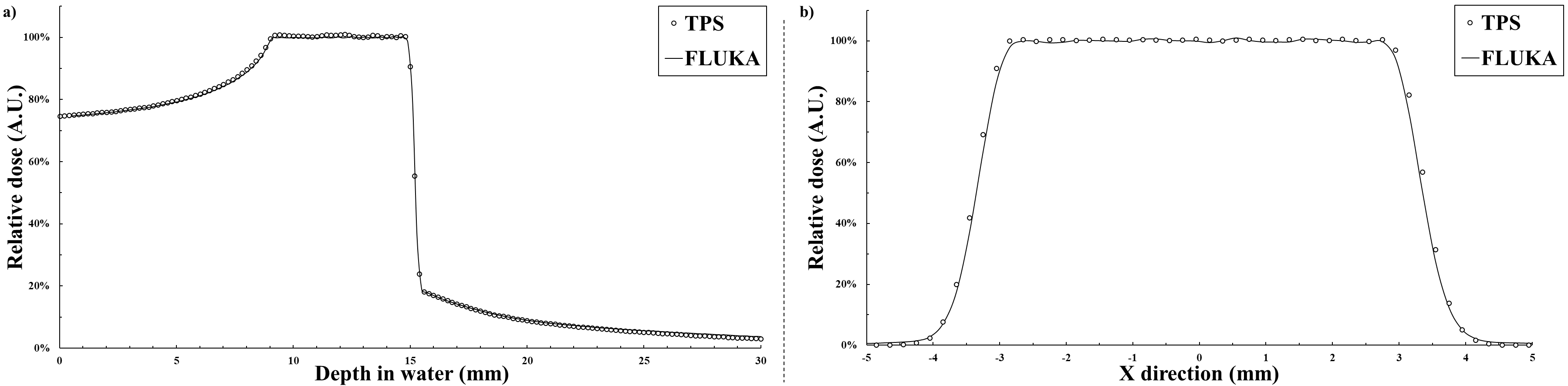


**Supplemental Fig 3** 1D dose profile comparison between the MC simulation (line) and TPS (dot) for M6CD12. Figure a is for the depth dose profile along the central axis, and figure b is for the transverse beam profile at the center of the cubes.


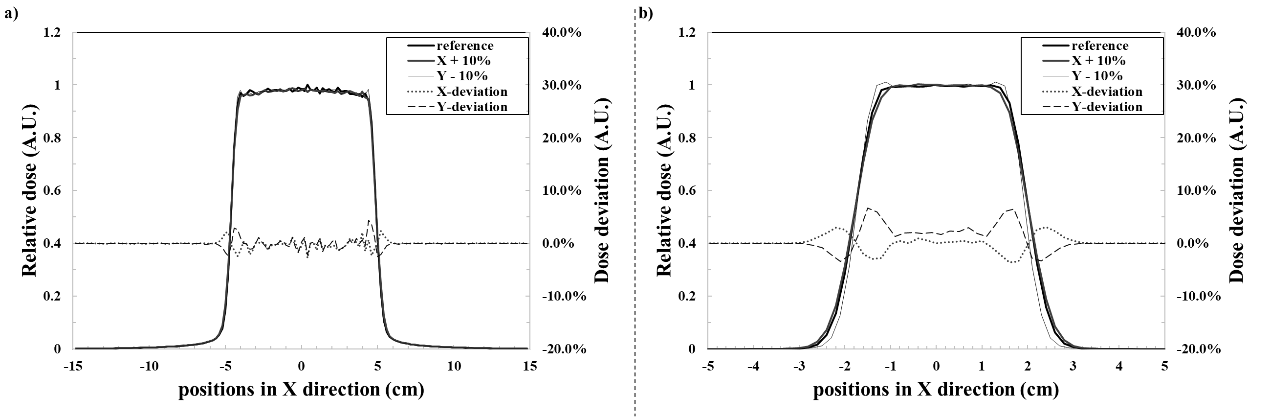


**Supplemental Fig 4** Relative dose (left axis) and dose deviation (right axis) on different positions in the cube M9CD24 (a) and the cube M3CD6 (b). Fine gray line represents 10% increase of spot size in the X-direction and the bold gray line represents 10% decrease of spot size in the Y-direction, the black line is the reference. Dotted and dash lines are represented to the deviations of X-direction and Y-direction, respectively.

**Supplemental TABLE Ⅰ**: The results of 3D absolute mean dose deviation, 2D γ-PRs (2%- 2 mm), the deviation of 1D flatness and lateral penumbra with spot sizes variation of +10% in X-direction and -10% in Y-direction

|  | 3D absolute mean dose deviation | 2Dγ-PRs | 1D lateral flatness deviation | | 1D lateral penumbra deviation/ mm | |
| --- | --- | --- | --- | --- | --- | --- |
|  |  |  | X（+10%） | Y（-10%） | X（+10%） | Y（-10%） |
| M3CD6 | 4.9% ± 6.5% | 97.6% | 2.8% | 0.7% | 1.8 | -1.5 |
| M3CD12 | 4.5% ± 5.1% | 97.5% | 2.3% | 1.7% | 1.6 | -1.1 |
| M3CD24 | 4.3% ± 5.1% | 99.5% | 2.1% | 1.3% | 1.4 | -1.1 |
| M6CD6 | 3.7% ± 5.8% | 97.9% | 2.0% | 0.3% | 1.5 | -1.3 |
| M6CD12 | 3.0% ± 4.8% | 98.3% | 2.5% | 3.9% | 1.6 | -1.2 |
| M6CD24 | 2.7% ± 4.2% | 98.2% | 1.3% | 0.5% | 1.3 | -1.4 |
| M9CD6 | 2.6% ± 4.1% | 98.3% | -0.7% | 3.7% | 1.3 | -1.1 |
| M9CD12 | 2.5% ± 3.9% | 98.5% | 2.0% | 2.3% | 1.3 | -1.2 |
| M9CD24 | 2.3% ± 3.4% | 99.9% | 0.9% | -0.2% | 1.2 | -1.2 |
